# Comparing the accuracy and precision of SMART1Map, SASHA and MOLLI

**DOI:** 10.1186/1532-429X-16-S1-P11

**Published:** 2014-01-16

**Authors:** Jeff A Stainsby, Glenn S Slavin

**Affiliations:** 1 GE Healthcare, Toronto, Ontario, Canada; 2 GE Healthcare, Bethesda, Maryland, USA

## Background

**Figure 1:**
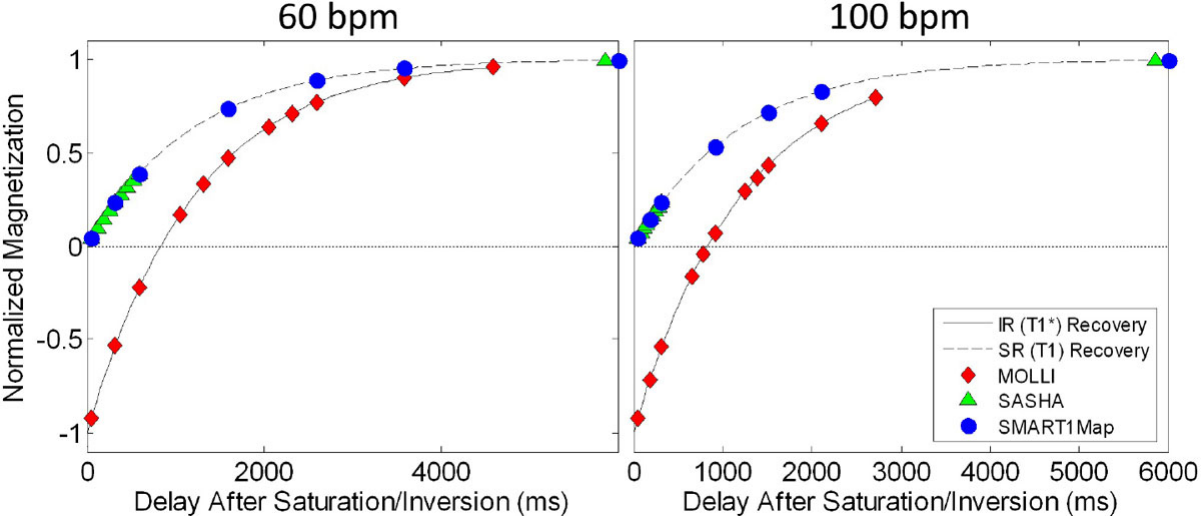
**Data sampling for SMAR1Map, MOLLI, and SASHA are illustrated for heart rates of 60 bmp (left) and 100 bpm (right)**. Sample points are shown on the saturation recovery curve ("SR" for SMART1Map and SASHA) or inversion recovery curve ("IR" for MOLLI) for a T1 (or T1*) of 1200 ms. SMART1Map and MOLLI obtain samples that are better distributed across the recovery curve, whereas SASHA is limited to many short sample times (<RR interval). At higher heart rates, the distribution of sample points for SASHA is further compressed. Note that both SMART1Map and SASHA acquire one "infitine" sample time with no preparation pulse, illustrated here at T1 = 6000 ms for convenience.

Currently, MOLLI [1] is the most common method for measuring myocardial T1. Because MOLLI uses an SSFP-based Look-Locker approach, only the apparent T1 (T1*) can be measured, and there are concerns about the accuracy of the T1* values due to the dependence on imaging parameters. Alternatives to Look-Locker imaging are single-point methods such as SASHA[2] and SMART1Map[3]. Although single-point methods are historically well-established and measure true T1, there remain differences between SASHA and SMART1Map that may affect their performance. Although both use single-point saturation recovery to acquire data at multiple saturation delay times (TS), the distribution of delay times is very different (Figure 1). The purpose of this work was to examine the accuracy and precision of MOLLI, SASHA, and SMART1Map in a phantom study.

## Methods

A phantom containing 22 samples of different T1s (200-1600 ms) and T1/T2 ratios (1.1 - 20.4) was imaged at 1.5T with MOLLI, SASHA, and SMART1Map using identical scan parameters. Scans were repeated 10 times each with simulated heart rates of 60 and 100 bpm. For SMART1Map and SASHA, T1 values were derived by curve fitting to A-B*exp[-TS/T1]. For MOLLI, A-B*exp[-TI/T1*] and the "Look-Locker correction" T1=(B/A-1)T1* were used. Reference T1s were determined with conventional single-point IR-spin echo.

## Results

**Figure 2:**
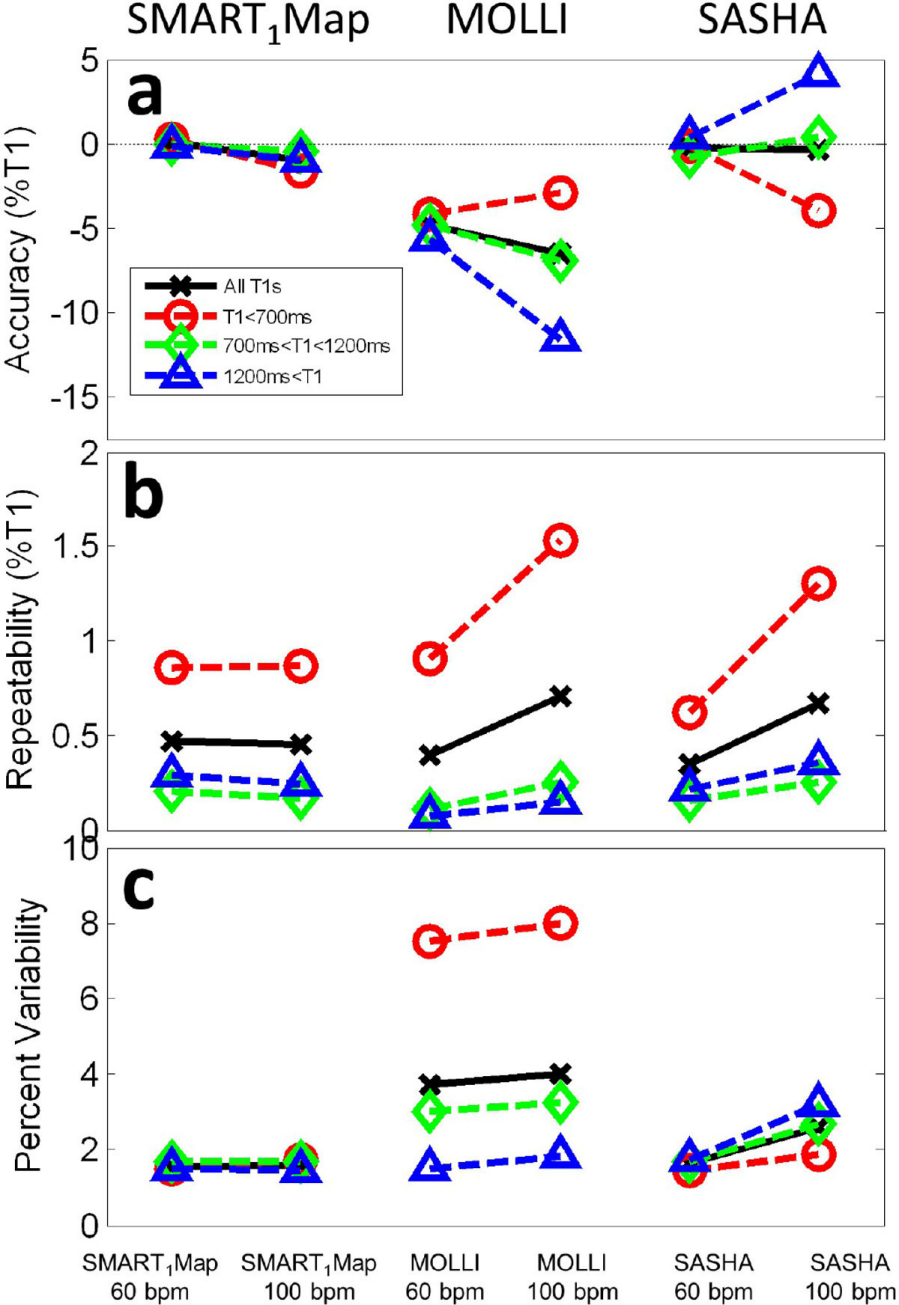
**Performance of SMART1Map (left), MOLLI (middle) and SASHA (right) at 60 and 100 bpm showing a) average accuracy of mean T1s over each of the 22 ROIs, b) repeatability of mean T1s over 10 repeats, and c) pixel-wise variability of measured T1 values within each sample**. All data are reported as a percentage of reference T1 values from IR-spin echo. Data are shown for 4 cases: **i**) averaged across all T1s (black line); **ii**) T1 < 700 ms (red, typical of post-contrast myocardium); **iii**) 700 < Y1 < 1200 ms (green); and **iv**) T1>1200 ms (blue, typical of native myocardium).

Results are summarized in Figure 2. As expected, SASHA and SMART1Map yield accurate estimates of true T1 overall, with MOLLI exhibiting the typical 5-10% underestimation and dependence on T1 and heart rate. The accuracy of SASHA degraded for longer T1s and higher heart rates. While all methods demonstrated generally repeatable results, both MOLLI and SASHA became less repeatable with increasing heart rates, whereas SMART1Map maintained high repeatability. The pixel-wise variability of T1 within each ROI was low in all cases with SMART1Map, while increased T1 variability was seen at shorter T1s with MOLLI and at higher heart rates with SASHA. Low variability in measured T1 is expected to be important for identifying small regions of pathology (e.g., for characterizing heterogeneous infarct).

## Conclusions

The sampling strategy of MOLLI yields low variability for long T1s and a high reproducibility, however the Look-Locker approach leads to high variability at short T1s and a low accuracy. The reduced accuracy of SASHA at longer T1s and higher heart rates is likely due to the limited distribution of sample times relative to the relaxation curve. This will pose a challenge for applying SASHA in systole (e.g., for right heart imaging), at higher heart rates, for non-contrast imaging, and at 3T, all of which further compress the range of sample times. Because SMART1Map has the unique ability to precisely measure delay times greater than one cardiac cycle, it provides the best combination of true T1 accuracy, high T1 repeatability and low T1 variability.

## Funding

N/A.
